# Single-cell profiling of cellular changes in the somatic peripheral nerves following nerve injury

**DOI:** 10.3389/fphar.2024.1448253

**Published:** 2024-10-02

**Authors:** Li Zhao, Chunyi Jiang, Bin Yu, Jianwei Zhu, Yuyu Sun, Sheng Yi

**Affiliations:** ^1^ Key Laboratory of Neuroregeneration of Jiangsu and Ministry of Education, Co-Innovation Center of Neuroregeneration, Nantong University, Nantong, China; ^2^ Department of Orthopedic, Affiliated Hospital of Nantong University, Nantong, China; ^3^ Department of Orthopedic, Nantong Third People’s Hospital, Nantong University, Nantong, China

**Keywords:** peripheral nervous system, peripheral nerve injury, dorsal root ganglion, sciatic nerve, single-cell sequencing

## Abstract

Injury to the peripheral nervous system disconnects targets to the central nervous system, disrupts signal transmission, and results in functional disability. Although surgical and therapeutic treatments improve nerve regeneration, it is generally hard to achieve fully functional recovery after severe peripheral nerve injury. A better understanding of pathological changes after peripheral nerve injury helps the development of promising treatments for nerve regeneration. Single-cell analyses of the peripheral nervous system under physiological and injury conditions define the diversity of cells in peripheral nerves and reveal cell-specific injury responses. Herein, we review recent findings on the single-cell transcriptome status in the dorsal root ganglia and peripheral nerves following peripheral nerve injury, identify the cell heterogeneity of peripheral nerves, and delineate changes in injured peripheral nerves, especially molecular changes in neurons, glial cells, and immune cells. Cell-cell interactions in peripheral nerves are also characterized based on ligand-receptor pairs from coordinated gene expressions. The understanding of cellular changes following peripheral nerve injury at a single-cell resolution offers a comprehensive and insightful view for the peripheral nerve repair process, provides an important basis for the exploration of the key regulators of neuronal growth and microenvironment reconstruction, and benefits the development of novel therapeutic drugs for the treatment of peripheral nerve injury.

## Introduction

The mammalian peripheral nervous system is an essential nerve system component that transmits bioelectrical signals throughout the body’s neural network. The peripheral nervous system, according to its anatomical, structural, and functional characteristics, is generally subdivided into sensory, motor (nerve fibers of motor neurons), enteric, and autonomic domains (Murtazina and Adameyko, 2023). The sensory nerves, together with axons of motor neurons, form the somatic peripheral nervous system (Catala and Kubis, 2013). The enteric nerves comprise myenteric and submucosal plexuses (Sasselli et al., 2012). The autonomic nerves are composed of the sympathetic system that dominates “fight or flight” emergency responses and the parasympathetic system that mediates physiology balance (Zahalka and Frenette, 2020). The cranial peripheral nervous system (cranial nerves III to XIII) have both somatic and autonomous fibers innervation. The cranial nerves III have both motor and parasympathetic fibers while the cranial nerves VII, IX, and X have motor, sensory, and parasympathetic fibers (Catala and Kubis, 2013).

The somatic peripheral nervous system comprises eight pairs of cervical spinal nerves, twelve pairs of thoracic spinal nerves, five pairs of lumbar spinal nerves, five pairs of sacral spinal nerves, and a few pairs of coccygeal nerves (Catala and Kubis, 2013). The somatic peripheral nerves are delicate and commonly affected by traumatic events such as wars, natural disasters, construction accidents, and traffic accidents. Peripheral nerves can also be affected by iatrogenic injuries such as surgical injuries and drug injection injuries. In addition, numerous diseases, such as diabetes, neurological disorders (for example, Guillain-Barre syndrome and carpal tunnel syndrome), and autoimmune diseases (for example, Sjogren’s syndrome, lupus, and rheumatoid arthritis), may result in injuries to peripheral nerve trunks. The characterization and functional investigations of somatic sensory nerves are more comprehensive as compared with those of enteric and autonomic nerves (Drokhlyansky et al., 2020).

**FIGURE 1:**
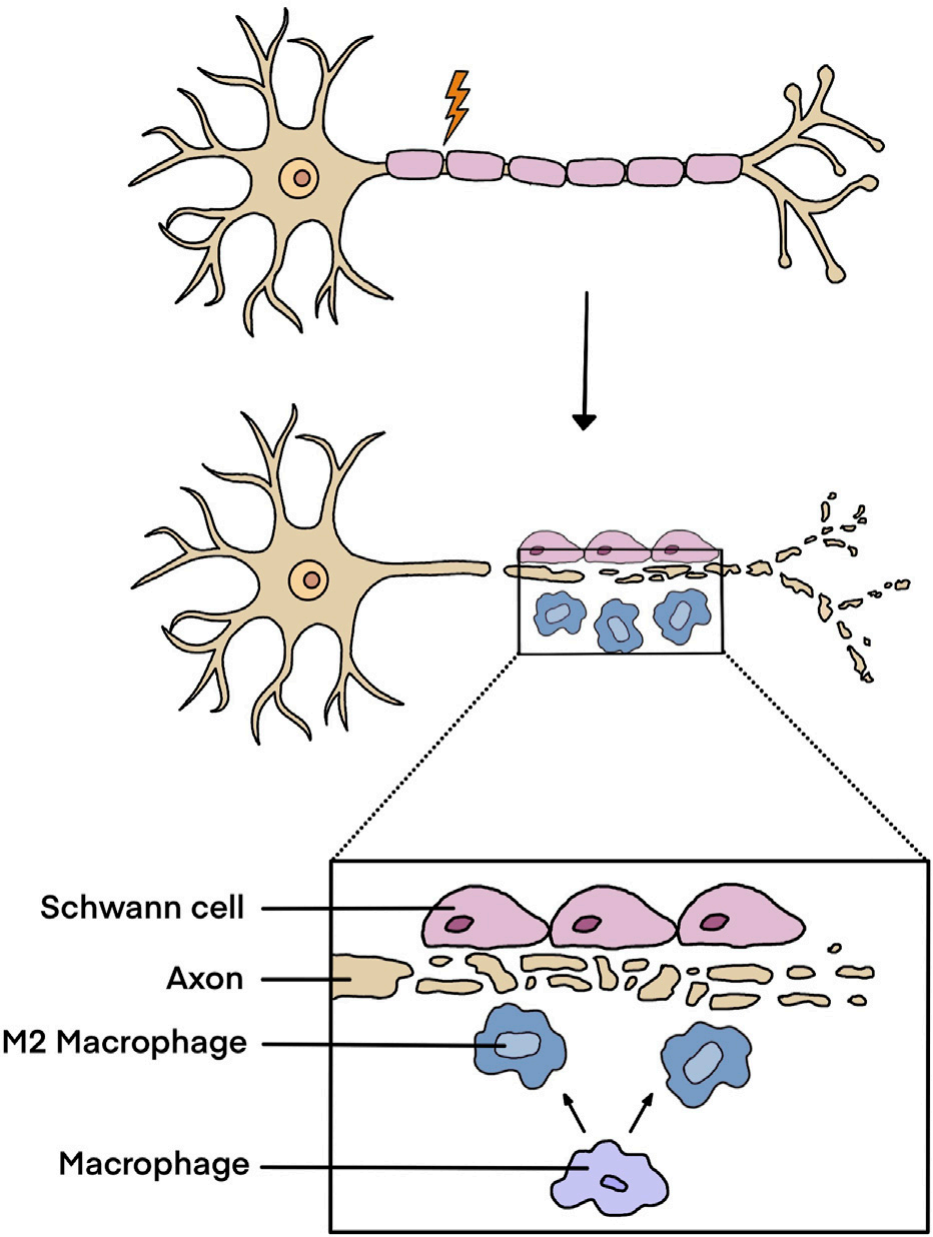
Injury responses in peripheral nerve stumps after peripheral nerve injury. Following peripheral nerve injury, Schwann cells and macrophages accumulate at the injured sites, clean axonal- and myelin-derived debris, and experience phenotype switch to generate a permissive microenvironment for nerve regeneration.

In peripheral nerve trunks, axons are wrapped with Schwann cells. Following peripheral nerve injury, especially those injuries with long nerve gaps, axon-associated Schwann cells undergo partial dedifferentiation and neuronal axons are transected. Afferent and efferent signals transmitted through neuronal axons are interrupted, inducing functional impairments, neuropathic pain, and physical disabilities. Peripheral nerve injury initiates acute immune responses within in nerve stumps and triggers inflammatory responses in neuronal somas (Kalinski et al., 2020). In both the proximal and the distal nerve stumps, numerous pro-inflammatory genes, such as *Il6*, *Cxcl3*, *Ccl20*, *S100a8/a9*, and *Mmp9*, are elevated and early immune responses are elicited (Chernov et al., 2015). Within 24–72 h after peripheral nerve injury, the axons distal to the injured sites and within a small zone distal to the proximal nerve stumps experience Wallerian degeneration, generating massive axonal- and myelin-derived debris. Schwann cells and immune cells are recruited into the injured sites to engulf and remove debris (Chen et al., 2015; Wong et al., 2017). Schwann cells sense injury signals, experience cellular reprogramming and are switched to a repair phenotype that benefits tissue homeostasis and regeneration via promoting the attraction of macrophages, the survival of neurons, and the elongation of axons (Jessen and Arthur-Farraj, 2019). Macrophages are polarized to an anti-inflammatory M2 phenotype to create a permissive microenvironment for nerve repair (Figure 1). Moreover, different from central nerves, in the peripheral nervous system, neurons retain regenerative ability following nerve injury (He and Jin, 2016). While distal axons undergo degeneration, axons at the proximal nerve stump are prepared for growth providing important basic for nerve repair and regeneration (Scheib and Höke, 2013). However, it is worth noting that although the peripheral nervous system obtains certain self-regeneration capacity, after long-distance peripheral nerve defect, peripheral nerve regeneration generally results in limited functional recovery without surgical and/or pharmacological treatments (Zou et al., 2023).

### Pharmacological treatments for peripheral nerve injury

Surgical suture is a typical method for the treatment of peripheral nerve injury (Meng et al., 2023). The joint application of therapeutic drugs helps to accelerate the regeneration process and boost the functional repair of injured nerves. Subcutaneous application of cyclic guanosine monophosphate-dependent phospodiesterase-5 inhibitor sildenafil to rats that undergo peripheral nerve transection and epineurial sutures reveals that sildenafil slightly improves rat sciatic functional index, although the changes of sciatic function index are not significant, the numbers of myelinated axons are not different, and the motor nerve conduction velocity are not affected (Fang et al., 2013). After rat peripheral nerve transection and subsequent incision site suture, the administration of serine protease inhibitor ulinastatin increases the expression of nerve growth factor (NGF), elevates the production of many myelin associated proteins, stimulates axon regeneration and nerve remyelination, and advances sciatic functional index as well as the amplitude of compound muscle action potential (Zhang et al., 2020).

Treatment with therapeutic drugs alone also benefits peripheral nerve regeneration, especially after relatively mild peripheral nerve injury such as nerve crush. Daily sildenafil treatment of sciatic nerve injured rats improves rat performance in the rotarod test (Korkmaz et al., 2016). Intraperitoneal injection of vitamin B complex containing B1, B2, B3, B6, and B12 to rats immediately after femoral nerve motor branch injury and every 24 h after surgery reduces the number/percentage of cells expressing pro-inflammatory cytokines and increases the number/percentage of cells expressing anti-inflammatory cytokines. The functional roles of vitamin B complex in local inflammation attenuation indicate that vitamin B can be used to treat peripheral nerve injury by reducing neuroinflammation (Ehmedah et al., 2019). The administration of immunosuppressant FK506 to rats undergo sciatic nerve crush injury directly reveals the promoting roles of immunosuppressive drugs in treating peripheral nerve injury. FK506 treatment accelerates the appearances of initial sign of return of toe movement and the initial return of ability to walk. Although serious quantification of myelin sheaths is not performed, morphological observations from light micrograph and electron microscopy show that daily subcutaneous injection of FK506 enhances nerve fiber myelination (Gold et al., 1994). Erythropoietin also exhibits immunomodulatory activity after peripheral nerve injury. Intraperitoneal injection of erythropoietin increases macrophage M2 activity, reduces in site apoptosis, bolsters debris phagocytosis, and benefits the myelination of nerve fibers as well as the recovery of sciatic functional index (Govindappa and Elfar, 2022). Co-treatment of erythropoietin with another anti-inflammatory drug dexamethasone further promotes the remyelination of injured peripheral nerves, stimulates functional recovery, and protects target muscle fibers from atrophy (Lee et al., 2020).

Current pharmacological treatments for peripheral nerve injury mainly take advantage of the anti-inflammatory effects of these drugs. Chronic inflammation may alter the behavior of cells in the nerve stumps, hamper the injury responses of Schwann cells, and diminish the regenerative capacity of injured nerves (Büttner et al., 2018). Hence, the application of drugs with anti-inflammatory and immunosuppressive properties may trigger the regeneration process. To identify novel therapeutic targets for the treatment of peripheral nerve injury, a recent study performs genome-wide CpG methylation profiling and identifies hypermethylation of formin-2 (fmn2) promoter as a key factor that mediates injury responses, microtubule dynamics at the growth cone, and axon regeneration. Next, by sequencing and *in silico* drug screen of fmn2 knockdown, small-molecule metaxalone has been discovered to be a regulator of microtubule dynamics and a novel therapeutic drug for the treatment of peripheral nerve injury (Au et al., 2023). Therefore, for the discovery of new therapeutic drugs, it is of great importance to determine genetic changes following peripheral nerve injury.

### Attempts to identify molecular changes in the peripheral nervous system after nerve injury

High-throughput analysis provides an unprecedented ability to explore molecular signatures under physiological and pathological conditions. Sciatic nerve is the thickest peripheral nerve truck in mammals. Hence, injury to sciatic nerves is commonly used for the investigation of peripheral nerve injury and regeneration (Savastano et al., 2014). Using the sciatic nerve experimental model, emerging high-throughput studies have examined large-scale molecular expressions following peripheral nerve injury and enumerated a large number of essential factors for nerve regeneration. A large number of microarray assays have determined the expression changes of both coding and non-coding genes in the dorsal root ganglia (DRGs), the proximal nerve stumps, and distal nerve stumps of injured sciatic nerves (Jia et al., 2020; Kubo et al., 2002; Li et al., 2015; Lu et al., 2014; Sohn and Park, 2020; Wang et al., 2018). Microarray has certain disadvantages, especially the limited detections of transcripts (Murakami et al., 2014). RNA sequencing approach has no such limitation and can even discover previously unknown transcripts and isoforms (Marioni et al., 2008; Soneson and Delorenzi, 2013). Actually, the application of RNA sequencing in the DRGs and nerve stumps after peripheral nerve injury has enabled the characterization of novel microRNAs and alternative splicing events (Li et al., 2012; Li et al., 2011; Zhao and Yi, 2019). The functional involvement of these transcripts, such as the promoting role of alternative splicing of neuronal cell adhesion molecule Nrcam in axonal growth and elongation, have been elucidated on the basis of RNA sequencing (Mao et al., 2018).

**FIGURE 2:**
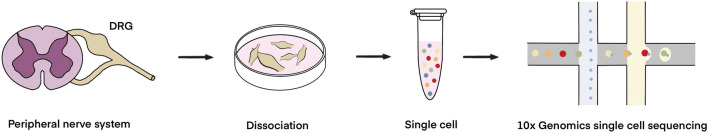
10 × Genomics single-cell sequencing workflow for the investigation of peripheral nerves. The DRGs and/or peripheral nerve stumps are dissociated into small pieces and then enzymatic treated to collect cell populations. Single-cell suspensions are dispensed through microfluidic partitioning and mixed with barcode-containing gel beads, reverse transcription reagents, and oil to obtain GEM for sequencing analysis.

Bulk sequencing generates transcriptome profiling by measuring averaged expressions of diverse constituent cells and thus masks specialized properties of single cells. Recently developed single-cell sequencing detects cell population heterogeneity and allows the identification of the states and functions of single cells (Tang et al., 2009). A series of single-cell sequencing technologies, ranging from targeted single-cell profiling to unbiased single-cell profiling, have been leveraged to study cellular population heterogeneity (Papalexi and Satija, 2018). Advances in droplet-based microfluidics push the development of massively parallel unbiased single-cell sequencing methods, such as indexing droplet sequencing (inDrop), Drop-seq, and 10X Genomics Chromium. The application of droplet-based microfluidics largely increases the number of cells in a single sequencing and decreases the cost for each cell (Zhang et al., 2019). These massively parallel unbiased sequencing technologies, especially the commercially available platform 10 × Genomics, have been widely used to decipher cellular populations in a variety of tissues and organs, including the peripheral nervous system (Nguyen et al., 2021; Xie et al., 2023; Zhang et al., 2022). For the 10 × Genomics analysis of the peripheral nervous system, tissues, such as the DRGs and peripheral nerve stumps, are processed to single-cell suspensions and functional gel-bead-in-emulsion (GEM) for dissecting cellular heterogeneity and detecting genetic expressions in specific cell types (Figure 2).

The single-nucleus sequencing method is also commonly used where a single nucleus is extracted, and nuclear transcriptomes are detected (Ding et al., 2020). Single-nuclei sequencing measures nuclear gene expressions and has been used as an alternative technology for optimal sample dissociation and cell storage (Denisenko et al., 2020). Compared with intact cells, a smaller amount of transcript is captured using single-nuclei sequencing because cytoplasmic information is lost. Still, single-nuclei sequencing has its own advantages and is extremely suitable for isolating cells from frozen tissues and formalin-fixed paraffin-embedded tissues as the freezing and fixation procedures interrupt cellular integrity and increase cytoplasmic transcript degradation (Cuevas-Diaz Duran et al., 2022). Single-nuclei sequencing is also more suitable to examine genetic features in cells with unusual morphologies and large sizes (Selewa et al., 2020). Some subtypes of neurons in the DRGs have large diameters and may not be able to successfully separated using microfluidic partitioning. For instance, the mean cell size of *Ptgfr*-expressing neuronal sub-type is reported to be 1,179 μm^2^ (Wang et al., 2023). Hence, it is appropriate to apply single-nuclei sequencing technology in the transcriptomic analysis of the DRGs as well as other neuron-containing tissues. Actually, single-nuclei sequencing has been frequently devoted to examine cellular heterogeneity in the peripheral nervous system (Avraham et al., 2022a; Yim et al., 2022). The application of these single-cell level analyses of the peripheral nervous system offers a wealth of knowledge of cellular and molecular processes under physiological and injury conditions.

### Single-cell-based analysis of the DRGs

DRG neurons possess certain intrinsic regeneration capacity and their regeneration capacity may be influenced by surrounding cells via cell-cell interaction and secreted factors. Single-cell sequencing, especially 10X Genomics, has been widely used to examine cellular populations in the DRGs under the naïve and injured states (Table 1).

**TABLE 1:** 10 × Genomics sequencing studies of DRGs.

Animal	Age	Cells	Clusters	Injury model	Time points	Refs
C57BL/6 mouse (female)	8–12 w	6541	13	sciatic nerve crush injury	3d	Avraham et al. (2020)
C57BL/6 mouse (female)	8–12 w	25,154	9	sciatic nerve crush injury, dorsal root crush injury, and spinal cord injury	3d	Avraham et al. (2021)
C57BL/6 mouse (female)	8–12 w	6343	11	-		Avraham et al. (2022b)
Lewis rat (male)	8–12 w	15,892	9	-		Avraham et al. (2022a)
C57BL/6J mouse (male)	7–8 w	36,810	9	spared nerve injury	6h, 24h, 2d, 7d, 14d	Wang et al. (2021b)

### Cellular population in the DRGs

Neuronal somas are surrounded by peripheral glial and other non-neuronal cells in the DRGs. Diverse types of cells have been identified in the DRGs (Table 2). Single-cell profiling of mouse L4 and L5 DRGs collects a total of 6541 cells in the naïve DRGs and the DRGs at 3 days after sciatic nerve crush injury, detects 1500 genes per cell, and categorizes cell populations into 13 cell clusters, i.e., blood, connective tissue, endothelial cells, macrophages, mesenchymal cells, neurons, neutrophils, pericytes, satellite glial cells, Schwann cells, vasculature associated smooth muscle cells, T cells, and an unidentified group (Avraham et al., 2020). These major cellular subtypes, including connective tissue, endothelial cells, macrophages, mesenchymal cells, neurons, pericytes, satellite glial cells, Schwann cells, smooth muscle cells, T cells, are also identified in a later study that sequences a total of 25,154 mouse DRG cells under normal condition and following peripheral and central branch injury (Avraham et al., 2021). These cell types are further detected in a single-cell sequencing in the L4 and L5 DRGs from rats and in a single-nucleus sequencing from the human DRG samples, showing high similarities between rodents and humans and indicating the translational applicability of rodent models in peripheral neuropathies (Avraham et al., 2022b). Some other cell types, including fibroblasts and distinct types of immune cells, such as B cells, are also captured (Renthal et al., 2020).

**TABLE 2:** Cellular populations in the DRGs identified by single-cell sequencing.

Animal	Method	Cell type	Refs
C57BL/6 mouse (both gender)	10x Genomics scRNA-seq	Blood, Connective tissue, Endothelial, Macrophages, Mesenchymal, Neurons, Neutrophils, Pericytes, Satellite glial cells, Schwann, Smooth muscle cells, T cells	Avraham et al. (2020)
C57BL/6 mouse (female)	10x Genomics scRNA-seq	N/A, T cells, Mesenchymal, Neurons, Schwann cells, Smooth muscle cells, Pericytes, Endothelial, Connective tissue, Macrophages, Satellite glial cells	Avraham et al. (2021)
C57BL/6 mouse (female)	10x Genomics scRNA-seq	Connective tissue, Endothelial, Macrophages, Mesenchymal, Neurons, Pericytes, Schwann cells, Satellite glial cells, Smooth muscle cells, T cells	Avraham et al. (2022b)
Lewis rat (male)	10x Genomics scRNA-seq	Schwann cells, Neurons, Macrophages, Endothelial, Connective tissue, Mesenchymal, Pericytes, Satellite glial cells	Avraham et al. (2022a)
Human (both gender)	snRNA-seq	Connective tissue, Endothelial, Macrophages, Mesenchymal, my Schwann cells, nm Schwann cells, Satellite glial cells, Smooth muscle cells, T cells	Avraham et al. (2022b)
C57BL/6 mouse (male)	InDrops snRNA-seq	Satellite glial cell, Schwann_M, Schwann_N, Macrophage, B cell, Neutrophil, Fibroblast, Endothelial, Pericyte	Renthal et al. (2020)
C57BL/6J mouse (male)	10x Genomics scRNA-seq	Neurons, Satellite glial cells, Schwann cells, Vascular endothelial cells, Vascular smooth muscle cells, Vascular endothelial cells capillary, Fibroblast, Immune cells, Red blood cells	Wang et al. (2021a)

Besides the recognition of cell type composition, single-cell sequencing also contributes to the categorization of neurons (Zeng and Sanes, 2017). Somatosensory neurons are traditionally classified to large diameter neurons (neurofilament-containing (NF) neurons) and small-medium diameter neurons (isolectin B4 (IB4)-positive non-peptide-containing (NP) neurons, peptidergic nociceptor-containing (PEP) neurons, and tyrosine hydroxylase-containing (TH) neurons) based on their morphological and molecular characteristics. These DRG neuron clusters have been discovered in a total of 622 neuronal cells in the mouse lumbar using single-cell tagged reverse transcription with the TH cluster occupies the largest population and the PEP cluster occupies the smallest population (Usoskin et al., 2015). Transcriptional profiling and functional assignment of single cells further allow the separation of low threshold mechanoreceptor (LTMR)-containing NF1-NF3 subpopulations and limb proprioceptive NF4 and NF5 subpopulations in the NF cluster, pruritus participating and itch sensing NP1-NP3 subpopulations in the NP cluster, thermosensitive PEP1 subpopulation and lightly myelinated Aδ nociceptor PEP1 subpopulation in the PEP cluster, and type C low-threshold mechanoreceptor TH neurons (Usoskin et al., 2015). A more detailed classification of DRG neurons has been obtained with high-coverage sequencing (Li et al., 2016; Wang K. et al., 2021). Comprehensive analyses of classification of DRG neurons have divided somatosensory neurons into a *Oprk1*-expressing sub-type, a *SStr2*-expressing sub-type, and a *Gabrg3*-expressing sub-type of small neurons for heat sensation, a *Trpm8*-expressing sub-type of small neurons for cold sensation, a *Rxfp1*-expressing sub-type of small neurons for cold sensation, a *Nppb*-expressing sub-type of small neurons, a *Mrgpra3*-positive and *Mrgpra4*-negative sub-type of small neurons, and two *Mrgprd*-expressing subtypes (*Lpar3*-positive or *Lpar3*-negative) of small neurons for itch sensation, two *Fam19a4*-expressing subtypes (*Th*-positive or *Th*-negative) of small neurons for c-LTMR sensation, a *Mrgpra3*-positive and *Mrgpra4*-positive sub-type of small neurons for message-like-stroking sensation, a *Wnt7a*-expressing sub-type of large neurons and a *Prokr2*-expressing sub-type of large neurons for proprioceptor sensation, a *Ptgfr*-expressing sub-type of large neurons for Aβ-LTMR sensation, a *Cox6a2*-expressing sub-type of large neurons for Aδ-LTMR sensation, as well as a Smr2-expressing sub-type of large neurons with undetermined function (Wang et al., 2023).

Non-neuronal cells, especially satellite glial cells, have also been grouped to diverse sub-types. Clustering of satellite glial cells from naïve DRG separates satellite glial cells to a *Pou3f1*-expressing sub-type that is associated with Kyoto Encyclopedia of Genes and Genomes (KEGG) pathways glycan biosynthesis and MAPK signaling, a *Gm13889*-expressing sub-type that is associated with cytokine and IL-17 signaling, a *Aldh1l1*-expressing sub-type that is associated with steroid biosynthesis and terpenoid backbone biosynthesis, and a *Scn7a*-expressing sub-type that is associated with ECM and cell adhesion pathways (Avraham et al., 2021).

### Phenotypic changes of DRG neurons in the DRGs after injury

Compared with the normal status, post injury changes are observed in neurons and other non-neuronal cells in DRG. Using 10X Genomics sequencing, in mouse L4 and L5 DRGs ipsilateral to the unilateral spared nerve injury, a surgical model that cuts the tibial and common peroneal nerve branches and induces alternations of neuronal firing activity and neuropathic pain, three new sub-types of neurons with high expressions of *Atf3*, including a *Atf3*/*Gfra3*/*Gal* sub-type, a *Atf3*/*Mrgprd* sub-type, and a *Atf3*/*S100b*/*Gal* sub-type, have been discovered from 24 h to 14 days after spared nerve injury. The *Atf3*/*Mrgprd*-labelled neurons peaks at 24 h post injury and rapidly disappears. This transiently existed neuron sub-type has high abundance of many regeneration-associated genes such as *Sox11*, *Sprr1a*, *Cacna2d1*, *Gadd45a*, and *Cckbr* and may switch to the *Atf3*/*Gfra3*/*Gal* neuron sub-type since 2 days post injury. The *Atf3*/*Gfra3*/*Gal* sub-type, as well as the *Atf3*/*S100b*/*Gal* sub-type, two neuron sub-types that are marked by axonal regeneration promoting neuropeptide galanin, are kept expressed at high levels in mouse DRGs from 2 days to 14 days after nerve injury and related with essential biological processes for nerve regeneration, such as inflammatory responses (Wang S. et al., 2021). Notably, the usage of spared nerve injury model to investigate DRG neuron changes after nerve injury may have some limitations as the spared nerve injury model induces partial denervation of one sciatic nerve branch and may affect other cell populations in the DRGs, including other connected neurons.

A recent single-cell sequencing study uses three different types of injury models, including L3 and L4 spinal nerve transection injury, sciatic nerve transection plus ligation injury, and sciatic nerve crush injury. A novel neuronal transcriptional state with high expressions of injury-induced genes *Atf3*, *Sprr1a*, *Sox11*, and *Flrt3*, is observed in all these injury models within 24 h after nerve injury (Renthal et al., 2020). A larger number of injured state neurons emerges after a more severe injury model. As high as 92.6% of neurons are recognized as the injured neuronal sub-type at 3 days after spinal nerve transection while only 41.4% of neurons switch to the injured state at the same time point after sciatic nerve transection plus ligation. These neurons at the injured state are capable to return to the native state at later time points after nerve injury and restore their original cell identify. Transcription factor ATF3, among high-expressed molecules in the injured neuronal sub-types, is found to be an indispensable driving factor for the transcriptional reprogramming of neurons (Renthal et al., 2020). Using an *in vitro* DRG neuron culture model, it is demonstrated that ATF3 facilitates DRG axon growth (Avraham et al., 2022a; Chandran et al., 2016). These findings validate previously observed injury-induced elevation of ATF3 in DRG neurons (Tsujino et al., 2000) and demonstrate that ATF3 is an important marker for neurons at the injured state. Besides *Atf3*, some other genes that are associated with neuronal repair process have been identified, such as *Adcyap1*, have been identified through exploring genetic changes in DRG neurons after nerve injury using experimental design single-cell RNA sequencing processing (Chen et al., 2023).

### Phenotypic changes of glial cells in the DRGs after injury

Satellite glial cells, glial cells that surround the soma of DRG neurons, exhibit diverse expression patterns with unique biological implications, especially fatty acid metabolism, at 3 days after sciatic nerve crush injury (Avraham et al., 2020). Conditional knockout of Fasn, a regulator gene of endogenous fatty acid synthesis, in satellite glial cells hinders axon regeneration while the inhibitory role of Fasn deletion in axon growth can be rescued by PPARα agonist fenofibrate. These genetic and functional findings fully illuminate that satellite glial cells respond to peripheral nerve injury and regulate neuronal actions (Avraham et al., 2020). A unique sub-type of satellite glial cells that is associated with Gene Ontology (GO) Molecular Function nervous system development and axon regeneration emerges in the DRGs at 3 days after sciatic nerve crush injury. Differentially expressed genes in this injury-specific satellite glial cell sub-type are enriched with transcription factor Zeb1, a regulator of epithelial to mesenchymal transition, as well as transcription factor Rest, a regulator of pluripotency. These findings indicate that peripheral nerve injury initiates the transition of satellite glial cells to a more plastic state (Avraham et al., 2021).

Compared with the well-elucidated genetic changes of Schwann cells at the injured site, transcriptional changes of Schwann cells in the DRGs has not been clarified until recently. Gene profiling demonstrates that Schwann cells localized in the DRGs also experience transcriptional changes at 3 days after sciatic nerve crush injury. Specifically, after peripheral nerve injury, myelination-promoting gene *Ngf* is downregulated and regeneration-regulating gene Yap is upregulated in Schwann cells localized in the DRGs (Avraham et al., 2021).

### Phenotypic changes of immune cells in the DRGs after injury

Injury-induced changes of immune cells in the DRGs, especially macrophages, have been explored as macrophages are implicated in neurite outgrowth and nerve regeneration (Niemi et al., 2013). Emerging studies demonstrate that macrophages not only infiltrate the injured peripheral nerve stumps but also accumulate at the DRGs (Zigmond and Echevarria, 2019). Besides the quantified enlarged cell number of macrophages in the DRGs, at 3 days post sciatic nerve injury, proliferation marker *Mki67* is highly expressed in macrophages and proliferation-associated pathways such as cell cycle and DNA replication are significantly enriched (Avraham et al., 2021). Moreover, in the DRGs, a rare sub-type of macrophages that non only express macrophage markers but also express satellite glial cell markers is found to be increased after nerve injury (Avraham et al., 2021). Similarly, another study also reports that macrophages with a stellate morphology and enveloping extensions exist in the axotomized DRGs instead of the DRGs in the naïve state from 24 h after sciatic nerve crush injury (Kalinski et al., 2020).

Many other non-neuronal cells in the DRG, besides glial cells and immune cells, also exhibit different biological features after peripheral nerve injury. For instance, the expressions of tight junction genes *Tjp1* and *Tjp2* in DRG endothelial cells are reduced at 3 days after sciatic nerve injury. Decreased expressions of barrier components in DRG endothelial cells in the injured state may impair blood-nerve barrier and increase permeability (Avraham et al., 2021).

### Single-cell-based analysis of peripheral nerves

As previously reported, sciatic nerve injury model is commonly used for the identification of cellular injury responses in the DRGs. Cellular and molecular changes in the injured peripheral nerves, especially injured sciatic nerves, are also commonly investigated to examine the wound environment after peripheral nerve injury (Table 3).

**TABLE 3:** 10 × Genomics sequencing studies of peripheral nerve stumps.

Animal	Age	Cells	Clusters	Tissue	Injury model	Time points	Refs
C57BL/6 mouse (both gender)	8–16 w	17,384	24	sciatic nerve	sciatic nerve crush injury	3d	Kalinski et al. (2020)
C57BL/6J mouse (female)	8–12 w	5400	12	brachial plexus and sciatic nerves	-	-	Wolbert et al. (2020)
C57BL/6J mouse (both gender)	P1, P60	11,339(P1), 12,428(P60)	11(P1), 9(P60)	sciatic nerve	-	-	Gerber et al. (2021)
C57BL/6 mouse (both gender)	8–16 w	21,973(naïve), 29,070(1d), 24,672(3d), 32,976(7d)	24(naïve), 22(1d), 24(3d), 34(7d)	sciatic nerve	sciatic nerve crush injury	naïve, 1d, 3d, 7d	Zhao et al. (2022)
C57BL/6 mouse (both gender)	8–16 w	17,404	13	sciatic nerve (injury or distal)	sciatic nerve crush injury	3d	Zhao et al. (2022)

### Cellular population in peripheral nerves

Peripheral nerve trunks are chiefly composed of fibroblasts, Schwann cells, and immune cells. A collection of 5400 viable cells from mouse brachial plexus and sciatic nerves is annotated to a variety of cell clusters using single-cell transcriptomes, including fibroblasts, Schwann cells, endothelial cells, T cells/natural killer cells, vascular smooth muscle cells, pericytes, macrophages, myeloid lineage cells, and endothelial cells, with Schwann cells and fibroblasts occupy 65% of the cell population (Wolbert et al., 2020). Schwann cells, vascular smooth muscle cells, pericytes, endothelial cells, and immune cells are also captured in another study that analyzes 4000 cells from mouse sciatic nerves. Fibroblasts and fibroblast-like cells or mesenchymal cells are alternatively recognized as *Pcolce2* and *Ly6c1*-expressing epineurial cells, *Itgb4* and *Slc2a1*-expressing perineurial cells, as well as *Col2a1* and *Sox9*-expressing endoneurial cells in this study (Gerber et al., 2021).

Schwann cells, especially Schwann cells surrounding peripheral axons, are commonly separated to myelinating and non-myelinating Schwann cells based on their morphological features. Myelinating Schwann cells form myelin sheathes wrapping neuronal axons and facilitate the rapid conduction of action potentials. The functional roles of non-myelinating Schwann cells are relatively less understood. The application of single-cell sequencing to peripheral nerves now allows the splitting of Schwann cells that express pan markers *Erbb3* and *S100b* to a myelinating sub-type that co-expresses myelin protein genes *Mbp*, *Mpz*, *Ncmap*, and *Plp1* as well as a non-myelinating sub-type that expresses *Ncam1* and *Ngfr*/p75. It is demonstrated that non-myelinating Schwann cells exist in a larger number than myelinating Schwann cells (Gerber et al., 2021; Wolbert et al., 2020). Notably, *Ncam1* and *Ngfr*/p75 are typical markers of immature Schwann cells, Schwann cells in late embryonic period and early postnatal period. Immature Schwann cells develop to myelinating and non-myelinating Schwann cells postnatally (Jessen and Mirsky, 2019). However, as far as we know, there is no direct transcriptional comparison between immature Schwann cells in embryos and non-myelinating Schwann cells in adult animals. The advanced genetic based separation of Schwann cell sub-types thus provides important molecular basis for the comparison between immature Schwann cells in embryos and non-myelinating Schwann cells as well as the investigation of the biological functions of non-myelinating Schwann cells.

### Phenotypic changes of Schwann cells in peripheral nerves after injury

Schwann cells in peripheral nerve stumps undergo remarkable cellular reprogramming. Both myelinating and non-myelinating Schwann cells switch to a repair phenotype with enhanced proliferation and migration capacity following nerve injury (Jessen and Mirsky, 2019). Schwann cells collected from sciatic nerve trunks that contains the injury site and the distal nerve stumps at 3 days after sciatic nerve crush injury are classified to three sub-types, including a *Mki67*-marked proliferating sub-type that expresses of *Ncam1*, *Cdl1*, *Erbb3*, *Epha5*, *Thbs2*, *Tnc*, *Hbegf*, and *Sostdc1*, a pro-myelinating-associated sub-type that expresses *Ngfr*/p75, *Nrcam*, *Gfra1*, *Btc*, *Gjb1*, *Cryab*, *Tnfrsf12a*, and *Gadd45b*, as well as a flanked sub-type with high expressions of *Nes*, *Cryab*, and genes coding for extracellular matrix such as *Bgn*, *Dcn*, and *Fn1* (Kalinski et al., 2020). However, due to a lack of tracing the trajectories of individual Schwann cells, the origins of these three Schwann cell sub-types are undetermined. It may be interesting to distinguish myelinating and non-myelinating Schwann cells and to explore the destination of myelinating and non-myelinating Schwann cells following nerve injury. In addition, the presences of immature Schwann cell marker *Ncam1* in *Mki67*-marked proliferating Schwann cell sub-type in the injured sciatic nerve stumps as well as the expression of immature Schwann cell marker *Ngfr*/p75 in pro-myelinating-associated Schwann cell sub-type imply that there may exist certain similarity between immature Schwann cells and repair Schwann cells. Another study investigates the expressions of Schwann cell markers in peripheral nerves at multiple stages of embryonic and postnatal development (E13.5, E17.5, P1, P5, P14, P24, and P60) and detects the high abundance of proliferating Schwann cell markers *Mki67* and *Top2a* as well as pro-myelinating Schwann cell marker *Ncam1* in developing sciatic nerves (E17.5) (Gerber et al., 2021). The identification of proliferating and pro-myelinating Schwann cell markers in embryonic mice further reveals certain transcriptional similarity between the development and the regeneration process.

### Phenotypic changes of immune cells in peripheral nerves after injury

The recruitment and infiltration of immune cells to the injured site are more vigorous as compared with the remote DRG tissues (Kalinski et al., 2020). The presence of immune cells, such as macrophages and neutrophils, contributes to debris removal during Wallerian degeneration, an essential preparatory stage for success regeneration (Lindborg et al., 2017; Zigmond and Echevarria, 2019). Single-cell sequencing of mouse sciatic nerves at the injured site and the distal nerve stumps at 3 days after sciatic nerve crush injury reveals that innate immune cells occupy approximately 44% of cell population and demonstrates the presence of T cells/natural killer cells, granulocytes, conventional dendritic cells, monocyte-derived dendritic cells, macrophages, and monocytes in sciatic nerves (Kalinski et al., 2020). Macrophages that express canonical markers *Adgre1*, *Aif1*, *Cd68*, and *Cx3cr1* are further classified to distinct sub-populations with diverse distributions. For instance, *Arg1* ^+^ macrophages are chiefly localized at the injured sites while F4/80^+^ macrophages are largely localized at the distal nerve stumps (Kalinski et al., 2020).

Steady-state sciatic nerve macrophages also demonstrate distribution-specific sub-populations. In the intact mouse sciatic nerves, a larger number of macrophages are *Relmα* ^−^ *Mgl*1^-^ *Iba-1* ^+^ macrophages and are localized inside the endoneurium of the sciatic nerve. A relative smaller number of macrophages are *Relmα* ^+^ *Mgl*1^+^ *Iba-1* ^+^ macrophages and are localized in the epineurium and the connective tissues surrounding nerve fascicles (Ydens et al., 2020). These two anatomically different sub-populations of macrophages exhibit dissimilar responses to peripheral nerve injury. Following peripheral nerve injury, epineurial *Relmα* ^+^ *Mgl*1^+^ resident macrophages do not sense injury signals while endoneurial *Relmα* ^−^ *Mgl*1^-^ macrophages are temporarily activated and can further be clustered to two sub-groups with differentially expressed genes enriched in canonical pathway granulocyte adhesion and diapedesis. Additionally, two novel clusters of macrophages, including a cluster of *S100a4*-expressing macrophages and a cluster of *H2-Aa*-expressing macrophages are found in the injured sciatic nerves at 1 day and 5 days after nerve injury, respectively. These emerged new macrophage sub-populations are considered to be newly recruited monocyte-derived macrophages and recruited macrophages that are further adapted to the wound microenvironment (Ydens et al., 2020). Single-cell sequencing of dissected the nerve injury site and distal nerve at 1 day, 3 days, and 7 days post sciatic nerve crush injury not only reveals the rapid accumulation of monocytes and macrophages in the injured nerve, but also demonstrates the metabolic reprogramming of immune cells, with monocytes and macrophages switch from a high glycolytic flux toward oxidative phosphorylation (Zhao et al., 2022).

Besides immune cells, structural cells in the peripheral nerve stumps also participate in shaping peripheral nerve inflammation. Mesenchymal progenitor cells are another main population in injured sciatic nerve stumps. Following sciatic nerve injury, mesenchymal progenitor cells express chemokines and display immune activity (Kalinski et al., 2020).

### Cell-cell interactions in peripheral nerves

Separating heterogenetic cell populations at the single-cell level fosters the understanding of intercellular communications. Script and software, such as CellChat (Jin et al., 2021), and CellPhoneDB (Efremova et al., 2020), quantify associations among ligands and receptors and thus infer interactions between different cell types and even sub-types. By using CellPhoneDB to interpret single-cell sequencing atlas of rat DRG, active ligand-receptor bindings among endothelial cells, fibroblasts, and vascular smooth muscle cells are found in neonatal rat DRG (Zhang et al., 2021). In neonatal rat sciatic nerves, close interactions between endothelial cells and fibroblasts as well as receptor-ligand pairs *a2b1* complex-*Col1a1*/*Col3a1* between endothelial cells are revealed, providing important genetic foundation for the discovery of innovative signal transductions (Zhang et al., 2021). Interestingly, although Schwann cells occupy a large cell population in peripheral nerve stumps, there are relatively few receptor-ligand pairs between Schwann cells and other cell types in intact sciatic nerves. Analyses of Drop-seq data of injured mouse sciatic nerves with Python script Cellcellinteractnet, combined with cell-surface mass spectrometry, predict considerable interactions of Schwann cells and endoneurial cells with axons (Toma et al., 2020). Paracrine interactions between surrounding cells with axons may thus support the growth and elongation of axons and accelerate peripheral nerve regeneration. An advanced bioinformatic tool Scriabin has been recently developed to analysis cell-cell interaction matrix at single-cell resolution without cell aggregation and therefore more single-cell based interaction features are expected (Wilk et al., 2023).

## Conclusion and perspective

The development of single-cell sequencing technologies and bioinformatic tools has enabled us to quickly obtain gene expression profiling of large numbers of individual cells and reveal the state and function of single cells. Herein, we summarize single-cell sequencing investigations of peripheral nerves, identify cellular and molecular hallmarks in the DRGs and peripheral nerve stumps in the native and injured states, and illuminate remote responses in axotomized DRGs and local response in injured peripheral nerve stumps. Still, it is worth mentioning that peripheral nerve injury also elicits changes in motor neurons whose neuronal somata are located in the spinal cord. The investigation of transcriptomic changes in motor neurons after peripheral nerve injury may contribute to the identification of potential strategies for the promotion of peripheral nerve regeneration, especially the recovery of motor functions.

Additionally, numerous cellular markers are either validated or newly-identified using single-cell sequencing. For instance, fatty acid binding protein gene *Fabp7*, a gene that is top enriched in the satellite glial cell cluster in the DRGs, clearly labels satellite glial cells enveloping neuronal soma, but does not label Schwann cells, and consequently is recognized as a novel specific marker for satellite glial cells (Avraham et al., 2020). Lipoprotein gene *Apod*, a gene that has been previously demonstrated to be present in Schwann cells, is expressed in a robustly higher abundance in non-myelinating Schwann cells as compared with myelinating Schwann cells and accordingly can be used for the identification of non-myelinating Schwann cells (Wolbert et al., 2020). Markers of peripheral cells are summarized and listed in Table 4. Improved algorithm software, such as OnClass, helps the categorization of cell populations and the identification of marker genes on a data-centric basis, regardless of the training data, and therefore may help paving the way for a more unseen cell types and sub-types (Wang K. et al., 2021).

**TABLE 4:** Marker genes for cellular populations in the somatic peripheral nerves.

Cell Type	Cell marker						
neuron	*Isl1*	*Tubb3*	*Gal*	*Tac*	*Prph*		
Schwann cell	*Pllp*	*Prx*	*Mag*	*Pmp2*	*Ncmap*		
myelinating Schwann cell	*Erbb3*	*S100b*	*Mbp*	*Pmp22*	*Mpz*		
non-myelinating Schwann cell	*Ngfr*	*P75*	*Cdh2*	*L1cam*	*Ednrb*	*Emp1*	*Sema3e*
satellite glial cell	*Apoe*	*Fabp7*	*Kcnj10*	*Kir4*	*Cdh19*	*Plp1*	
fibroblast	*Apod*	*Fn1*	*Fgfr1*	*Col1a*	*Col3a*		
endothelial cell	*Pecam1*	*Cd31*	*Flt1*	*Cldn5*	*Ly6c1*	*Plvap*	*Esam*
macrophage	*Aif1*	*Iba1*	*Cd68*	*Cx3cr1*			
mesenchymal cell	*Cd34*	*Apod*	*Pdgfra*				
T cell	*Cd3g*	*Cd3e*					
B cell	*Cd79*	*Cd19*					
erythrocyte	*Hba*	*Hbb*					
pericyte	*Kcnj8*	*Pdgfrb*	*Rgs5*				
connective tissue	*Col1a1*	*Dcn*					
vascular smooth muscle cell	*Myocd*	*Acta2*	*Des*	*Pln*	*Tpm2*	*Tagln*	

As a recently developed technology, single-cell sequencing technology still has some limitations (Garcia-Flores et al., 2023; Mutisheva et al., 2022). Complete separation of cell populations and acquisition of single cells are initial steps that fundamentally affect the precision of sequencing data. Take rodent sciatic nerve tissues, for example, it generally takes hours from tissue collection to cell suspension loading. Extended time may impact the quality of isolated cells and induce inaccurate outcomes. Proper enzyme digestion methods and single-cell separation procedures are thus critical for gene expression profiling. Another weakness is that it is hard to obtain a consistent and comparable cellular types and sub-types among different single-cell sequencing data due to inaccurate measurement, differently employed marker genes, and diverse clustering. Improved sequencing technologies and bioinformatic tools as well as enlarged recognition of marker genes, especially marker genes for rare cells, may help to solve the existing problem and offer matched data for molecular characterization and biological implication (Pullin and McCarthy, 2024).

Taken together, a better understanding of the peripheral nerve heterogeneity during the peripheral nerve injury and regeneration process benefit the cognition of essential molecules and novel cell sub-populations that regulate peripheral nerve regeneration and hence may contribute to the discovery new therapeutic drugs targeting these molecules and cell sub-populations.
